# Quantitative MRI in Neuroimaging: A Review of Techniques, Biomarkers, and Emerging Clinical Applications

**DOI:** 10.3390/brainsci15101088

**Published:** 2025-10-08

**Authors:** Gaspare Saltarelli, Giovanni Di Cerbo, Antonio Innocenzi, Claudia De Felici, Alessandra Splendiani, Ernesto Di Cesare

**Affiliations:** Department of Biotechnological and Applied Clinical Sciences, University of L’Aquila, 67100 L’Aquila, Italy; giovanni.dicerbo@graduate.univaq.it (G.D.C.); antonio.innocenzi@graduate.univaq.it (A.I.); claudia.defelici@graduate.univaq.it (C.D.F.); alessandra.splendiani@univaq.it (A.S.); ernesto.dicesare@univaq.it (E.D.C.)

**Keywords:** quantitative MRI, neuroimaging, imaging biomarkers, diffusion MRI, perfusion imaging, brain volumetry

## Abstract

Quantitative magnetic resonance imaging (qMRI) denotes MRI methods that estimate physical tissue parameters in units, rather than relative signal. Typical readouts include T1/T2 relaxation (ms; or R1/R2 in s^−1^), proton density (%), diffusion metrics (e.g., ADC in mm^2^/s, FA), magnetic susceptibility (χ, ppm), perfusion (e.g., CBF in mL/100 g/min; rCBV; K^trans^), and regional brain volumes (cm^3^; cortical thickness). This review synthesizes brain qMRI across T1/T2 relaxometry, myelin/MT (MWF, MTR/MTsat/qMT), diffusion (DWI/DTI/DKI/IVIM), susceptibility imaging (SWI/QSM), perfusion (DSC/DCE/ASL), and volumetry using a unified framework: physics and signal model, acquisition and key parameters, outputs and units, validation/repeatability, clinical applications, limitations, and future directions. Our scope is the adult brain in neurodegenerative, neuro-inflammatory, neuro-oncologic, and cerebrovascular disease. Representative utilities include tracking demyelination and repair (T1, MWF/MTsat), grading and therapy monitoring in gliomas (rCBV, K^trans^), penumbra and tissue-at-risk assessment (DWI/DKI/ASL), iron-related pathology (QSM), and early dementia diagnosis with normative volumetry. Persistent barriers to routine adoption are protocol standardization, vendor-neutral post-processing/QA, phantom-based and multicenter repeatability, and clinically validated cut-offs. We highlight consensus efforts and AI-assisted pipelines, and outline opportunities for multiparametric integration of complementary qMRI biomarkers. As methodological convergence and clinical validation mature, qMRI is poised to complement conventional MRI as a cornerstone of precision neuroimaging.

## 1. Introduction

Conventional magnetic resonance imaging (MRI) refers to the imaging techniques routinely used in clinical practice to describe pathology by analyzing contrast differences in weighted images [1,2,3,4,5]. These images predominantly, though not exclusively, reflect biophysical contrast mechanisms, such as T1- and T2-weighted scans [6,7,8,9,10].

Over the past decade, significant advancements have occurred in the field of MRI, including innovations in hardware, pulse sequence design, image reconstruction techniques, and data analysis algorithms [11,12,13,14,15]. These technological improvements have shifted attention toward quantitative MRI (qMRI), which aims to derive objective metrics from MR images that are directly related to specific physical or biophysical tissue properties [13,16,17,18].

Quantitative imaging provides insight into biological processes by measuring parameters that may serve as biomarkers, rather than relying solely on relative signal intensities with arbitrary units, as in routine diagnostic imaging [13,19,20,21]. Thus, qMRI techniques offer superior sensitivity to subtle abnormalities in both lesions and normal-appearing tissue and can improve specificity by identifying the nature of tissue damage [2,22,23,24].

However, for quantitative biomarkers to be reliable and clinically useful, the acquisition and analysis protocols must be standardized. Kessler et al. defined a quantitative imaging biomarker (QIB) as “an objective characteristic derived from an in vivo image measured on a ratio or interval scale, serving as an indicator of normal biological processes, pathological processes, or response to therapeutic intervention.” Unlike conventional imaging, where sensitivity and specificity describe diagnostic power, QIBs must demonstrate technical performance in terms of bias (accuracy), precision (variability), and linearity to ensure reliable use in diagnosis, monitoring, and prognosis [3,25,26,27,28,29].

Currently, qMRI is not widely adopted in clinical practice because most techniques have not reached clinical maturity [16,30,31,32]. A qMRI method is considered clinically mature when it can be implemented on standard clinical scanners without the need for custom pulse sequences, with validated software for data analysis and interpretation, and established diagnostic cut-off values [33,34,35,36].

## 2. Scope and Organization of the Review

This review focuses on adult brain quantitative MRI (qMRI) across neurodegenerative, neuro-inflammatory, neuro-oncologic, and cerebrovascular conditions. To facilitate navigation and comparability, each modality is presented using a substantially uniform framework—including Physics and signal model, Acquisition and key parameters, Outputs and units, Validation and repeatability, Clinical applications, Limitations and pitfalls, Future directions—in the following fixed order: T1 relaxometry (R1), T2 relaxometry and myelin-water/magnetization transfer, diffusion (DWI/DTI/DKI), susceptibility imaging (SWI/QSM), perfusion (DSC/DCE/ASL), and volumetry. Topics such as cortical plasticity/learning-related plasticity, pediatric/developmental trajectories, and rehabilitation-induced remodeling are acknowledged but fall outside the primary scope of this article. This review focuses on imaging-based quantitative MRI modalities; MR spectroscopy is outside the scope and therefore not covered. A disease-centric summary linking major qMRI biomarkers to specific clinical conditions is provided in Table 1. Alongside acquisition and post-processing harmonization, we emphasize standardization of output visualization—including units, scale limits, report layout, and color conventions—so that T1/T2, QSM, rCBV/K^trans^, ASL-CBF, and volumetric outputs can be consistently interpreted across scanners and sites [31,32].

## 3. Search Strategy and Selection Criteria

We searched PubMed/MEDLINE, Scopus, and Web of Science for studies published 1 January 2000–1 October 2025. Queries combined “quantitative MRI/qMRI” with modality terms (T1/T2 relaxometry; MT/MWF; diffusion—DWI/DTI/DKI/IVIM; susceptibility—SWI/QSM; perfusion—DSC/DCE/ASL; volumetry) and clinical contexts (multiple sclerosis, dementia/Alzheimer’s, stroke, glioma). **Inclusion**: peer-reviewed human brain studies, consensus/standards, methodological/validation (including phantoms), and systematic reviews/meta-analyses; adult populations unless the modality is inherently developmental. **Exclusion**: non-quantitative MRI, non-brain works unless informing standardization, single-case reports without quantitative endpoints, and non-English. Records were deduplicated, screened by title/abstract, and full texts assessed; reference lists were hand-searched. When overlaps occurred, we prioritized consensus/validation and multicenter/multivendor reproducibility, then representative clinical studies. Last update: 1 October 2025.

## 4. Biological and Clinical Ground Truth for qMRI Validation

Beyond technical performance (bias, precision, linearity), translation requires linking qMRI metrics to biological and clinical ground truth. Biological validation includes post-mortem MRI–histology co-registration (e.g., myelin/iron stains vs. R1/MWF/QSM), biopsy-level or segment-wise correlations, and—where tissue is not feasible—alignment with orthogonal biomarkers (e.g., PET, CSF) [39,40]. Clinical validation spans cross-sectional discrimination, longitudinal responsiveness (including minimal clinically important differences, MCID), and prognostic/predictive value for outcomes (disability, relapse, survival). These studies must be underpinned by test–retest, Bland–Altman, and multicenter/multivendor repeatability, ideally following consensus profiles (e.g., QIBA), and supported by physical/digital phantoms and open protocols for pre-registration and power planning.

## 5. Visualization and Interpretability of qMRI Maps

Clinical adoption of qMRI depends not only on technical performance but also on how metrics are visualized and read. We therefore recommend: (i) perceptually uniform colormaps with explicit units (e.g., T1/T2 in ms, χ in ppm, ADC in mm^2^/s, CBF in mL/100 g/min); (ii) consistent scale limits across timepoints and patients to enable longitudinal and cross-subject comparisons; (iii) anatomical overlays and linked multi-panel views (e.g., structural T1 with parametric map) for spatial context; (iv) reference distributions (e.g., regional histograms/z-scores) for interpretability; (v) optional uncertainty or quality control maps (fit residuals, SNR, motion metrics) to flag unreliable voxels. These practices complement standardized acquisition and processing and are intended to lower the threshold for radiologist adoption.

A cross-modality summary of acquisition requirements, approximate scan times, primary outputs (with units), strengths, and common limitations is provided in Table 2.

## 6. Positioning Relative to Broader qMRI Reviews

Cross-domain qMRI surveys span multiple organ systems and provide broad methodological overviews. In contrast, our review is domain-focused on the adult brain and integrates method physics, validation/repeatability, standardized outputs, and clinical use-cases (neurodegenerative, neuro-inflammatory, neuro-oncologic, cerebrovascular), aiming at near-term clinical translation. We view these approaches as complementary: cross-domain breadth provides context, whereas domain-specific depth is required to specify clinically actionable pipelines.

## 7. T1 Relaxometry

### 7.1. Physics and Signal Model

T1 relaxometry measures the recovery of longitudinal magnetization of excited spins in tissue, providing quantitative T1 relaxation time values. These values are sensitive to both microstructural and macrostructural tissue integrity, particularly myelin, axonal density, and water content [1]. In practice, after an inversion or saturation pulse, the longitudinal magnetization returns toward equilibrium with a time constant T1; most clinical methods assume a mono-exponential recovery [41,42,43,44,45]. Apparent T1-relaxation time can be biased by B1 inhomogeneity, incomplete inversion/saturation, and magnetization transfer (MT), so flip-angle (B1) correction and MT-aware processing are recommended; inversion-recovery approaches tend to be more accurate, whereas variable-flip-angle methods are faster but more B1-sensitive [41,42,43,46,47]. Exchange between free-water and myelin-bound pools and partial volume effects further explain deviations from ideal behavior [48,49,50,51,52,53]; for reporting, some studies also provide R1 (=1/T1) alongside T1-relaxation time. In multiple sclerosis (MS), several studies have demonstrated that T1-relaxation time correlates strongly with both myelin and axon content in normal-appearing white matter (NAWM) and white matter lesions [1,54].

In white matter, exchange between free-water and myelin-bound pools leads to non-mono-exponential longitudinal recovery; routine clinical fits are therefore mono-exponential approximations that yield an apparent T1 dominated by the free-water pool and influenced by MT and partial volume. This should be considered when comparing across sequences/sites; where feasible, IR-based methods (with B1 and MT-aware corrections) and complementary metrics (R1, MTsat) can improve interpretability and reduce bias [41,42,43,44,45,46,47].

### 7.2. Acquisition and Key Parameters

From a technical standpoint, T1 mapping can be achieved using different acquisition strategies, including inversion recovery (IR), saturation recovery (SR), variable flip angle (VFA), and hybrid methods such as Look-Locker, MOLLI, or SAPPHIRE. Each technique offers specific trade-offs between scan time, accuracy, and sensitivity to artifacts. For example, IR-based methods provide gold-standard accuracy but are time-consuming, while VFA methods are fast but highly sensitive to B1 inhomogeneity. Accurate T1 quantification also depends on correcting for MT effects, B1 field variations (e.g., actual flip-angle imaging or Bloch–Siegert shift), and partial volume contamination in cortical or small white matter regions [46,47].

### 7.3. Outputs and Units

**Figure 1:**
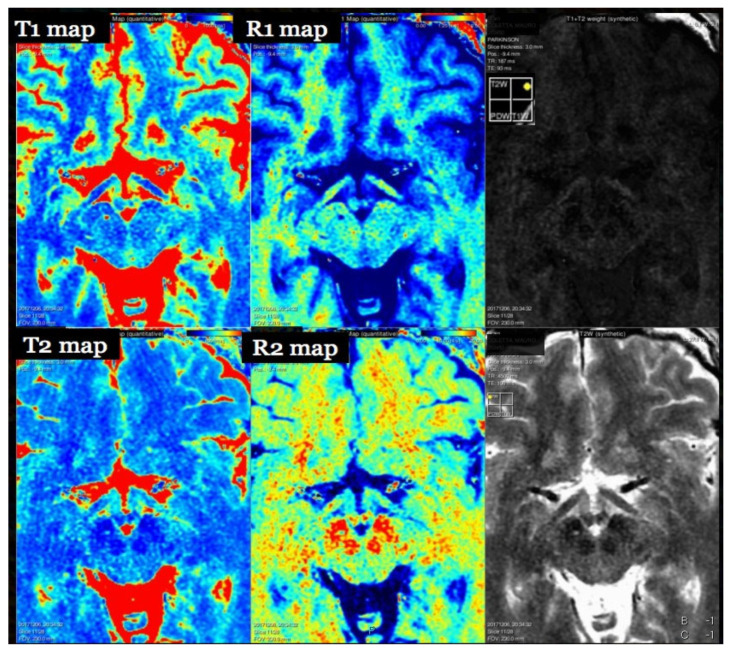
Quantitative parametric maps and synthetic contrasts from a single qMRI acquisition. Representative brain slice showing T1 map (ms), R1 map (s^−1^; R1 = 1/T1), T2 map (ms), and R2 map (s^−1^; R2 = 1/T2) (left and middle panels), alongside synthetic contrast–weighted images reconstructed from the quantitative data (right panels; example T1 + T2-weighted and T2-weighted). As expected, CSF exhibits long T1/T2 (high values on T1/T2 maps; correspondingly low R1/R2), white matter shows shorter T1/T2 (higher R1/R2), and gray matter is intermediate; iron-rich deep nuclei tend to have elevated R2. These maps enable region-wise statistics (e.g., histograms), longitudinal monitoring (ΔT1/ΔT2/R1/R2), and on-the-fly synthesis of conventional contrasts by selecting TE/TR/TI in post-processing. Color scales are arbitrary but consistent across maps for visualization.

Various quantitative biomarkers can be derived from T1 relaxometry. In addition to absolute T1-relaxation time values, metrics such as:

- R1 = 1/T1, a linear proxy for myelin content,
- T1-normalized intensity, typically normalized to CSF or gray matter for inter-subject comparison,
- Histogram-based features (mean, standard deviation, skewness, kurtosis) of T1 values in NAWM,
- ΔT1 values for longitudinal lesion monitoring,

have been proposed as potential biomarkers for disease activity, progression, or treatment response [41,42,43,44,45,46,47]. Several studies have shown that T1 histogram metrics in NAWM predict disability progression and correlate with cognitive impairment in MS [54]. Representative whole-brain T1 and R1 maps—and the corresponding T2/R2 maps with synthetic contrast-weighted images—are shown in Figure 1.

### 7.4. Validation and Repeatability

Nonetheless, promising approaches have emerged. One such technique is the MP2RAGE sequence (Magnetization Prepared 2 Rapid Acquisition Gradient Echoes), which enables highly reproducible T1-relaxation time maps with a coefficient of variation as low as 3% [41,42,43,44,45]. MP2RAGE has been validated in phantom and multicenter studies using 3T MRI scanners, demonstrating excellent intra- and inter-scanner repeatability [30,55,56,57]. An alternative and increasingly popular method is synthetic MRI (SyMRI), which simultaneously acquires quantitative maps of T1, T2, and proton density (PD), along with a B1 correction map. These maps (R1, R2, PD) represent intrinsic MR tissue properties and can be used to synthetically generate multiple contrast-weighted images from a single ~6 min scan, with flexible adjustment of echo time (TE), repetition time (TR), and inversion time (TI) [6,56,57,58,59,60]. Reproducibility across scanners and sites remains limited, particularly in multivendor environments [45,61,62,63].

### 7.5. Clinical Applications

Prolonged T1-relaxation time is associated with demyelination, axonal loss, and iron depletion, reflecting more destructive tissue changes and extracellular water accumulation. Conversely, T1 shortening or stabilization over time may indicate reparative processes such as remyelination or gliosis. As such, quantitative T1 metrics can act as surrogate biomarkers to distinguish between active, chronic, or reparative lesion stages.

T1 relaxometry has significant relevance in developmental neuroimaging. During CNS myelination—most rapid in the first two years of life and continuing through adolescence—T1 and T2 relaxation times decrease, while anisotropy and MT metrics increase. This maturation process reflects structural reorganization and water compartmentalization in white matter, making T1 mapping a potential quantitative biomarker in pediatric neurology. T1-derived growth curves have been used to assess normal brain maturation and detect early deviations in neurodevelopmental disorders, such as hypoxic–ischemic injury, metabolic encephalopathies, or autism spectrum disorder [64,65,66].

### 7.6. Multimodal Integration

T1 relaxometry also benefits from multimodal integration. When combined with diffusion tensor imaging (DTI), magnetization transfer imaging (MTI), or quantitative susceptibility mapping (QSM), T1 mapping can improve tissue characterization by offering complementary information. For instance, the combination of R1 and MTR better distinguishes between demyelination and inflammation, while integration with QSM helps characterize iron-related changes and microstructural damage. To coherently fuse R1 with MTR/MTsat and diffusion/QSM metrics at the patient level, AI-driven post-processing (co-registration, denoising, outlier-aware harmonization, and composite visualization) is increasingly necessary [67,68].

### 7.7. Limitations and Pitfalls

Despite its diagnostic potential, T1 relaxometry is not yet widely implemented in routine MS clinical protocols. Several technical and methodological challenges persist:

1. Multiple competing acquisition methods exist, each with varying sensitivity to confounding factors such as magnetization transfer (MT), B1 inhomogeneities, diffusion, and T2 effects [41,42,43];
2. No consensus has been reached on the optimal sequence for accurate in vivo T1 mapping [44];
3. T1 relaxation in white matter is known to follow bi-exponential behavior due to magnetization exchange with myelin-bound protons, while most available methods assume mono-exponential decay [45];
4. Reproducibility across scanners and sites remains limited, particularly in multi-vendor environments [61,62,63,69].

## 8. T2 Relaxometry and Magnetization Transfer

### 8.1. Physics and Signal Model

Quantitative T2 relaxometry (qT2) estimates tissue water content by fitting a mono-exponential signal decay, capturing contributions from both intracellular/extracellular water and myelin-associated water. More advanced models, such as multi-component T2 relaxometry, allow the separation of distinct water pools [70,71,72,73,74]—most notably the myelin water fraction (MWF), which quantifies the proportion of water trapped between myelin bilayers and serves as a surrogate marker of myelin integrity. These models rely on multi-echo sequences and fitting algorithms such as non-negative least squares (NNLS) or Bayesian inference to resolve the fast-decaying myelin water component (typically <40 ms) from intra/extracellular water (70–90 ms) and cerebrospinal fluid (>200 ms) [70,71,72,73,74]. In practice, the mono-exponential assumption is an approximation: refocusing flip-angle imperfections and stimulated-echo pathways, B1/B0 inhomogeneity, and echo spacing influence the observed decay [46,47]. Accurate qT2 therefore benefits from multi-echo spin-echo (or GRASE) acquisitions with refocusing control and, when needed, fat suppression, plus forward models (e.g., EPG-based) that account for slice profile and stimulated echoes [46,47,70,71,72,73,74]. For multi-component qT2, the inversion is ill-posed and requires regularization/priors (as in NNLS/Bayesian approaches); MWF estimates also depend on noise floor, partial volume, and potential exchange between pools [48,50,51,52,53,70,71,72,73,74]. Documenting TE train length, number of echoes, and echo spacing alongside T2/MWF outputs improves reproducibility and interpretability [70,71,72,73,74].

### 8.2. Acquisition and Key Parameters

Several acquisition strategies have been developed to estimate MWF in clinically feasible timescales, including multi-echo spin-echo, gradient-and-spin-echo (GRASE), and multi-component DESPOT (mcDESPOT) [70,71,72,73,74,75,76,77]. Recent advancements, such as compressed sensing and parallel imaging, have reduced acquisition times to under 6 min while maintaining accuracy [46,47]. Furthermore, histogram analysis of qT2 values can provide additional microstructural insight by capturing tissue heterogeneity and subtle demyelination through changes in distribution metrics such as standard deviation or skewness [70,71,72,73,74,78,79].

### 8.3. Magnetization Transfer (MT) Framework

Magnetization transfer (MT) imaging complements qT2 by selectively saturating protons bound to macromolecules (such as myelin), enabling the derivation of semi-quantitative metrics like the magnetization transfer ratio (MTR) and MT saturation (MTsat). While MTR is straightforward to compute, it is influenced by acquisition parameters and B1 field inhomogeneity. MTsat offers improved specificity by accounting for T1 dependence [52,80] and flip-angle variability. More advanced quantitative MT (qMT) models represent tissue as a two-pool system [51,52,53] (free and bound proton pools), enabling the estimation of additional parameters such as the macromolecular pool fraction (f), exchange rate (k), and T2 of the bound pool [48,49,50].

### 8.4. Clinical Applications

In multiple sclerosis (MS), MTR abnormalities have been detected in normal-appearing white matter (NAWM) prior to the appearance of gadolinium-enhancing lesions. MTR has also shown dynamic longitudinal changes that correspond to demyelination and remyelination, especially in subpial cortical regions and the spinal cord of patients with progressive disease. Lower MTR values have been observed in cervical cord regions in both relapsing-remitting and primary progressive MS, with progressive decline over time [59,79]. Similar trends have been observed with MTsat and MWF, which have shown greater specificity for myelin loss and potential for detecting reparative changes. Several longitudinal studies have demonstrated increases in MWF or MTsat in response to treatment, suggesting their potential as biomarkers of remyelination and tissue repair [81,82].

### 8.5. Validation and Repeatability

Although qT2, MWF, and MT imaging are not yet part of routine clinical protocols, recent technical advances have enabled acquisition of qT2 and MWF maps in 3–6 min, which may be compatible with clinical workflow. Preliminary fast-acquisition protocols for spinal cord MWF mapping have also emerged. However, clinical implementation remains limited, particularly for MTR reconstruction, due to the lack of standardized and validated sequences across vendors. In addition, partial volume effects and variability due to scanner hardware and field strength continue to challenge widespread adoption [75,76,77].

### 8.6. Multimodal Integration and Future Directions

As with other quantitative imaging biomarkers—such as diffusion (DWI) or perfusion imaging (DCE)—the future adoption of qT2 and MT metrics in personalized medicine will depend on demonstrating robust repeatability and reproducibility, especially in multicenter and longitudinal studies [16,30]. Biomarkers derived from these modalities—such as MWF, MTsat, and qMT parameters—are currently under evaluation in several multicenter initiatives [75]. Their integration with other quantitative measures, such as diffusion tensor imaging (DTI) or quantitative susceptibility mapping (QSM), may provide complementary insights into demyelination, axonal injury, and inflammation. In addition, the use of MWF in pediatric imaging shows promise for tracking myelination during development and identifying early neurodevelopmental disorders [77].

## 9. Diffusion Imaging (DWI, DTI, DKI)

### 9.1. Physics and Signal Model

Diffusion-weighted imaging (DWI) is a cornerstone of modern clinical MRI, particularly essential for the early diagnosis of acute ischemic stroke, and widely used in the assessment of brain tumors and intracranial infections. It captures the random Brownian motion of water molecules, which is hindered by biological structures such as cell membranes and organelles. The apparent diffusion coefficient (ADC), calculated from a mono-exponential model, quantifies average water diffusivity in tissue and is reported in mm^2^/s [1,83]. In practice, diffusion weighting is encoded with pulsed gradient spin-echo (Stejskal–Tanner–type) preparations; the degree of weighting is governed by the “b-value,” which increases with gradient amplitude, duration, and separation. Under the Gaussian (mono-exponential) assumption, higher b-values produce greater signal attenuation and the ADC summarizes ensemble-averaged diffusivity; deviations from mono-exponential behavior at higher b-values reflect microstructural restrictions and heterogeneity [84].

Diffusion tensor imaging (DTI) expands upon DWI by adding directional information, modeling diffusion as an ellipsoid rather than a sphere. This captures anisotropic diffusion, especially prominent in white matter tracts where water movement is constrained along axonal pathways. In tensor terms, diffusion is represented by a 3 × 3 symmetric positive-definite matrix whose eigenvalues/eigenvectors encode principal diffusivities and orientations; rotationally invariant scalars (e.g., FA, MD, AD, RD) are derived from these eigenvalues [3,4]. Accurate tensor estimation requires sufficient unique gradient directions and appropriate b-values [55,85], alongside correction for motion and eddy-current distortions and mitigation of susceptibility-induced EPI warping [83,84].

Diffusion kurtosis imaging (DKI) is an extension of DTI that accounts for non-Gaussian diffusion, which arises due to complex tissue environments [86]. The apparent kurtosis coefficient (AKC) quantifies this deviation, offering increased sensitivity to microstructural complexity and heterogeneity [87,88]—especially relevant in stroke, tumors, and neurodegeneration [83]. Practically, reliable DKI estimation uses multiple b-values (often up to ~2000–2500 s/mm^2^) and dense angular sampling to stabilize the higher-order fit [89,90,91].

Common confounders across DWI/DTI/DKI include bulk motion and physiological pulsatility, eddy currents, gradient nonlinearity, susceptibility-induced EPI distortions, Gibbs ringing, Rician noise-floor bias, CSF partial volume, and B0/B1 inhomogeneity. Standard remedies include motion/eddy correction, distortion correction (e.g., field-mapping or reversed-phase encoding), denoising and ringing suppression, fat suppression and shimming, and careful ROI/segmentation to minimize partial volume effects [91].

### 9.2. Acquisition and Key Parameters

DWI typically uses three orthogonal diffusion directions and fast 2D multi-slice acquisitions. Recent advances include the use of high b-values (e.g., b = 2000–3000 s/mm^2^) to improve sensitivity to restricted diffusion, especially in highly cellular tumors and acute stroke. However, at these values, diffusion decay becomes non-monoexponential, motivating the use of higher-order models such as DKI or IVIM. Multi-band accelerated EPI and reduced field-of-view DWI are increasingly used to reduce susceptibility artifacts and improve resolution, especially in spinal cord imaging [55].

For DTI, advanced acquisition protocols recommend ≥30 diffusion directions for robust tensor estimation, and newer approaches like high angular resolution diffusion imaging (HARDI) are being explored to mitigate the limitations of the single-tensor model in regions with crossing fibers. While HARDI improves depiction of complex fiber configurations, it entails longer acquisitions and lower SNR at higher b-values [85]; estimates depend on b-value/shell design and response-function assumptions (for deconvolution), and remain sensitive to motion, eddy currents, and susceptibility-induced EPI distortions [1,83,84]. Orientation distribution functions (ODFs) reflect orientation rather than specific microstructural parameters [85]; partial volume with CSF, gradient nonlinearity, and lack of standardized pipelines further limit cross-site comparability [16,30]. Clinical interpretation should therefore emphasize robust scalar derivatives and quality-assured tractography rather than over-interpreting model-specific parameters [3,4,55].

Compared to DTI, DKI requires higher b-values (up to 2500 s/mm^2^) and more diffusion directions, but provides better differentiation of tissue types in gray matter, tumors, and ischemic penumbra. In stroke, MK often exceeds the spatial extent of the DWI lesion and may capture tissue at risk [1,92]. In neuro-oncology, increased MK and RK have been correlated with tumor grade, cellularity, and microenvironmental heterogeneity [88,93,94].

### 9.3. Outputs and Units

From DTI, the principal rotationally invariant scalar metrics include:

- Fractional anisotropy (FA): degree of diffusion directionality,
- Mean diffusivity (MD): average diffusivity, equivalent to ADC but derived from tensor data,
- Axial diffusivity (AD): diffusion parallel to axons,
- Radial diffusivity (RD): diffusion perpendicular to axons [3,4].
- From DKI, key parameters include:
- Mean kurtosis (MK): overall measure of tissue complexity,
- Axial kurtosis (AK): non-Gaussianity along the primary fiber axis,
- Radial kurtosis (RK): kurtosis perpendicular to axonal direction, sensitive to myelin integrity.
- In addition, NODDI provides two parameters:
- Neurite density index (NDI): reflects axonal and dendritic density,
- Orientation dispersion index (ODI): measures angular variation in neurites.
- In neuro-oncology, ADC has proven valuable in:
- Grading gliomas (high vs. low grade),
- Distinguishing gliomas from metastases,
- Differentiating tumor progression from pseudo-progression, and
- Predicting IDH mutation status and survival [9,95,96].

Although ADC is influenced by acquisition protocol and tumor heterogeneity, multiple meta-analyses confirm its utility as a quantitative imaging biomarker. The QIBA diffusion profile suggests that longitudinal ADC changes ≥11% reflect true biological variation [10,16,97,98].

### 9.4. Clinical Applications

DTI is widely used to assess white matter integrity in conditions such as multiple sclerosis (MS), stroke, spinal cord injury (SCI), and brain tumors. In MS, DTI-derived abnormalities—such as decreased FA and increased RD—have been associated with demyelination, axonal injury, and cognitive or physical disability. Longitudinal DTI studies have demonstrated microstructural changes in both lesions and normal-appearing white matter that correlate with clinical progression [11,22,99,100,101,102,103,104].

In SCI, DTI metrics can detect tissue damage at the lesion site and in adjacent spinal cord segments. Acute SCI shows decreased FA and AD and increased RD, suggesting both axonal and myelin damage. These metrics correlate with functional outcomes and are sensitive to degeneration above and below the lesion, often not visible on conventional MRI [100,105,106].

In degenerative cervical myelopathy (DCM), studies show that reduced FA and increased MD at the stenosis level—and even rostrally—correlate with subclinical degeneration and functional impairment. These changes are detectable even in asymptomatic individuals with cord compression [107,108,109,110,111].

**Figure 2:**
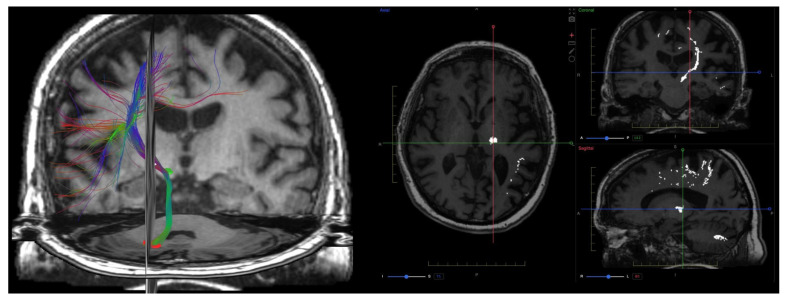
Multimodal integration of diffusion tractography and volumetric segmentation on structural MRI (presurgical planning example). (**Left**): Streamline tractography of major perilesional white-matter pathways (color-coded by local orientation) overlaid on a high-resolution T1-weighted image, illustrating the spatial relationship between fibers and the abnormality. (**Center/right**): Lesion/target segmentation mask (white) co-registered to the same T1 reference and displayed in axial, coronal, and sagittal planes. This combined view supports risk assessment and trajectory planning by showing fiber displacement/encasement relative to the lesion while providing volumetric measurements for longitudinal follow-up. Color legend: tractography streamlines are RGB direction-encoded—red = left–right, green = anterior–posterior, blue = superior–inferior; the anatomical background is grayscale; the lesion/target mask is white.

In neuro-oncology, patient-specific DTI tractography complements volumetric lesion segmentation by depicting displacement or encasement of eloquent white-matter pathways relative to the tumor, informing risk assessment and surgical planning (Figure 2).

### 9.5. Validation and Repeatability

Standardization, repeatability, and validation of diffusion metrics are crucial for clinical translation and integration into personalized medicine and treatment monitoring. The QIBA diffusion profile suggests that longitudinal ADC changes ≥11% reflect true biological variation [10,16,97,98].

### 9.6. Emerging Techniques and Integration

Newer diffusion models such as neurite orientation dispersion and density imaging (NODDI) and vascular, extracellular, and restricted diffusion for cytometry in tumors (VERDICTs) aim to disentangle complex microstructural compartments (e.g., intracellular, extracellular, and vascular spaces). These models improve tissue specificity but currently remain within research settings [55,85]. In brain development and neurodegeneration, NODDI-derived metrics have shown greater sensitivity than DTI to changes in neurite architecture and may serve as future imaging biomarkers.

Advanced spinal DTI techniques incorporating reduced-FOV EPI and motion correction have improved the reliability of these measures.

### 9.7. Summary and Outlook

Diffusion MRI techniques provide non-invasive biomarkers of tissue microstructure, applicable across a wide range of neurological disorders. While DWI and DTI are clinically established, DKI and emerging compartmental models offer enhanced sensitivity and specificity, particularly in MS, oncology, and spinal cord pathology.

## 10. Quantitative Susceptibility Mapping (QSM) and Susceptibility-Weighted Imaging (SWI)

### 10.1. Physics and Signal Model

Magnetic susceptibility reflects the ability of a material to become magnetized in the presence of an external magnetic field [112]. This property has become a powerful endogenous source of tissue contrast in MRI, particularly through Susceptibility-Weighted Imaging (SWI) and Quantitative Susceptibility Mapping (QSM). In gradient-echo (GRE) acquisitions, microscopic susceptibility differences perturb the local magnetic field and produce phase shifts that grow with echo time; SWI exploits this by combining magnitude with high-pass–filtered phase (typically at longer TEs) to enhance venous structures, microbleeds, iron, and calcifications, yielding a semi-quantitative contrast sensitive to local susceptibility variations.

QSM, in contrast to SWI, reconstructs quantitative, voxel-wise maps of tissue magnetic susceptibility from the phase data of GRE sequences [113,114,115]. The process involves: (i) estimating the local magnetic field perturbation from the phase images; (ii) removing background fields (e.g., from air–tissue interfaces); (iii) solving the inverse problem to deconvolve the field shifts with a dipole kernel, allowing the direct computation of tissue susceptibility. These steps are implemented using pipelines that include phase unwrapping, background field removal (e.g., SHARP, RESHARP, or PDF), and dipole inversion with algorithms such as TKD, iLSQR, or MEDI. Regularization techniques, like Morphology Enabled Dipole Inversion (MEDI), are crucial to solving the ill-posed dipole inversion problem [116]. In practice, multi-echo 3D GRE improves SNR and phase linearity; careful masking, echo combination, and handling of air–tissue interfaces help minimize artifacts. QSM yields tissue-specific values in ppm (paramagnetic vs. diamagnetic), whereas SWI provides a highly sensitive but semi-quantitative visualization.

**Figure 3:**
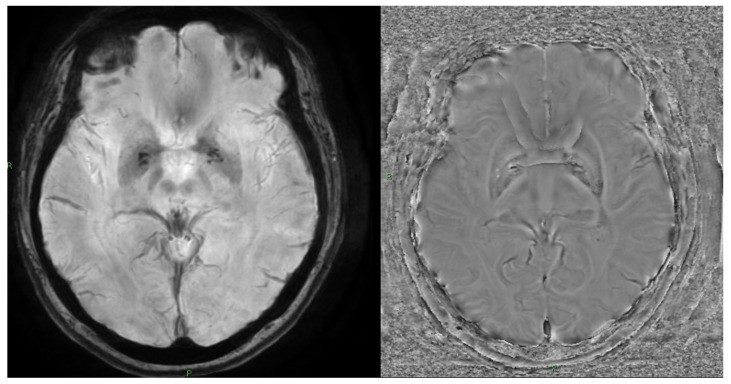
SWI magnitude versus QSM from the same GRE acquisition. (**Left**): Susceptibility-weighted imaging (SWI) magnitude image (long-TE GRE) highlighting venous structures and focal paramagnetic deposits as hypointense foci due to susceptibility-induced blooming. (**Right**): Quantitative Susceptibility Mapping (QSM) reconstructed from the GRE phase (after phase unwrapping, background-field removal, and dipole inversion), providing voxel-wise χ values (ppm) with reduced blooming and improved tissue specificity. Paramagnetic sources (e.g., veins/iron-rich regions) appear with positive χ, whereas diamagnetic sources (e.g., calcium) would show negative χ, enabling differentiation of calcification from hemorrhage and facilitating longitudinal quantification.

SWI emphasizes susceptibility-related blooming on the magnitude image, whereas QSM reconstructs voxel-wise χ (ppm) with reduced blooming and improved tissue specificity (Figure 3).

### 10.2. Acquisition and Key Parameters

SWI is a gradient-echo (GRE)-based technique that combines magnitude and filtered phase images—typically using long echo times—to enhance contrast arising from differences in local magnetic susceptibility [9,69]. The resulting susceptibility-weighted images, often visualized through minimum intensity projections (mIPs), are particularly sensitive to paramagnetic substances such as deoxyhemoglobin, hemosiderin, and ferritin, enabling improved visualization of venous vasculature, microhemorrhages, calcifications, and iron deposits in the brain [2,99,110].

QSM acquisition typically employs 3D multi-echo GRE sequences to enhance SNR and improve phase linearity over time. This yields quantitative measures of paramagnetic (e.g., iron, deoxyhemoglobin) and diamagnetic (e.g., calcium, myelin) tissue components [117].

### 10.3. Outputs and Units

A key distinction is that SWI is a semi-quantitative visualization technique, while QSM provides numerical, tissue-specific metrics expressed in parts per million (ppm). Compared to T2*-weighted imaging, QSM offers more accurate tissue characterization by reducing blooming artifacts and geometric distortions [48,50,117]. This makes QSM more suitable for longitudinal tracking and cross-subject comparisons [118].

### 10.4. Clinical Applications

Multiple Sclerosis (MS). QSM has shown superior sensitivity in detecting early and chronic MS lesions. Increased susceptibility values in active lesions correspond to myelin breakdown and iron accumulation within macrophages. Chronic rim-positive lesions—defined by a hyperintense peripheral rim on QSM—have been associated with slowly expanding lesions and smoldering inflammation, which correlate with disease progression and poor prognosis [119,120]. When combined with relaxation mapping (e.g., T1, T2) or magnetization transfer metrics (e.g., MTsat), QSM allows improved differentiation between iron-related pathology and myelin loss [117,121].

Traumatic Brain Injury (TBI). SWI and QSM outperform diffusion imaging in detecting microhemorrhages and small-vessel injuries, particularly in diffuse axonal injury. QSM has also demonstrated greater diagnostic sensitivity than fractional anisotropy in mild TBI by capturing tissue damage not limited to axonal membranes but also involving iron-related pathology and myelin integrity [117,122,123,124,125].

Cerebral Microbleeds and Vascular Pathologies. SWI and QSM are considered gold standards for visualizing cerebral microbleeds (CMBs) due to their high sensitivity to hemosiderin. QSM enables artifact-free quantification of lesion burden and allows for the distinction between hemorrhage (paramagnetic) and calcification (diamagnetic) [126,127], which appear similarly hypointense on conventional GRE images. Moreover, QSM can estimate venous oxygen saturation and oxygen extraction fraction (OEF), with potential application in ischemic stroke, hypoperfusion, and arteriovenous malformations [83,117,128].

Neuro-Oncology. In brain tumors, QSM improves the ability to distinguish hemorrhagic deposits from calcifications [129] and may aid in characterizing intratumoral vasculature and neovascularization. Elevated susceptibility in tumor rims may reflect hemorrhagic necrosis or iron deposition associated with angiogenesis. QSM is being explored as a biomarker for treatment response in gliomas and recurrent glioblastomas [122], particularly in the context of anti-angiogenic therapies [117,129].

Spinal and Extra-CNS Applications. While QSM is not yet routinely used in spinal imaging, both QSM and SWI have been explored in evaluating spinal cord hemorrhage, disk degeneration, and carotid atherosclerotic plaques [130,131]. In carotid imaging, SWI can distinguish intraplaque hemorrhage (paramagnetic) from calcification (diamagnetic) based on polarity of susceptibility. These features may offer diagnostic value in assessing asymptomatic carotid disease or spinal cord injury, although susceptibility artifacts from adjacent bone and air remain a technical challenge [110,111,132].

### 10.5. Limitations and Pitfalls

Despite its promise, clinical adoption of QSM faces technical and logistical challenges:

- Lack of standardization among reconstruction algorithms and no universally accepted processing pipeline;
- Offline post-processing requirements that are complex and time-consuming;
- Limited vendor integration, although standard GRE sequences used for SWI or T2* can often be repurposed for QSM if phase images are preserved.

QSM reconstruction often prioritizes numerical stability over image contrast, leading to potential oversmoothing and reduced visibility of fine anatomical details. Nevertheless, multiple studies have demonstrated excellent intra- and inter-scanner reproducibility in both healthy controls and MS patients, supporting QSM’s technical robustness as a quantitative imaging biomarker [117,133].

The dipole kernel has zeros on a conical surface in k-space, making inversion ill-posed and amplifying noise/streaking [113,114,115]. Regularization choices balance stability vs. accuracy: TKD (k-space thresholding) is fast but yields underestimation and orientation bias, iLSQR/Tikhonov provide stable solutions at the cost of smoothing, and MEDI/TV-based approaches use morphological priors to preserve edges but can oversmooth fine detail if over-regularized [113,114,115,116]. Parameter selection (e.g., L-curve, cross-validation) and uncertainty mapping help document confidence, while COSMOS (multi-orientation) offers a reference standard but is impractical clinically [113,114,115,116,134].

### 10.6. Future Outlook

Efforts such as the QSM Challenge and the development of open-source toolkits (e.g., MEDI, STI Suite, QSMxT) are helping to improve standardization and reproducibility across research sites [134]. As reconstruction algorithms become faster and more clinically integrated, QSM is expected to play a growing role in neurodegenerative, vascular, oncological, and spinal imaging, offering tissue-specific, quantitative insights that go beyond conventional MRI contrast mechanisms [17]. Building on the QSM Challenge and open-source pipelines (MEDI, STI Suite, QSMxT), the next step is clinically integrated software that preserves fine anatomical detail while providing uncertainty maps and vendor-agnostic reporting, an area where AI-assisted regularization selection and artifact rejection can bridge current gaps [134].

## 11. Perfusion Imaging

### 11.1. Physics and Signal Model

Perfusion imaging plays a central role in assessing the delivery of blood—and thus oxygen and nutrients—to tissues, providing critical insights into microvascular function and tissue viability [135]. In magnetic resonance imaging (MRI), perfusion can be evaluated using either exogenous tracers (gadolinium-based contrast agents, GBCA) or endogenous tracers (magnetically labeled arterial blood water in ASL) [136]. Among perfusion techniques, two dynamic contrast-enhanced MRI methods are primarily employed: Dynamic Susceptibility Contrast (DSC) and Dynamic Contrast-Enhanced MRI (DCE). Both rely on serial imaging acquisitions before, during, and after intravenous administration of gadolinium, but they differ in imaging sequences, contrast mechanisms, and physiologic parameters derived.

**Dynamic Susceptibility Contrast (DSC).** In DSC, a T2*-weighted EPI sequence captures the first pass of a GBCA bolus: susceptibility-induced microscopic field gradients around intravascular contrast cause a transient signal drop proportional to the local contrast concentration [2,90,137,138]. Using an arterial input function (AIF) and the indicator-dilution framework, rCBV is obtained from the area under the tissue concentration–time curve (normalized by the AIF), while rCBF arises from deconvolution of tissue and arterial curves; MTT follows from the central-volume principle [83,139,140]. Accurate quantification depends on short TR, appropriate TE, and high temporal resolution to sample the bolus peak [2,83]. A key confound is contrast leakage through a disrupted blood–brain barrier, which introduces T1 effects into the T2*-weighted signal; mitigation includes pre-bolus/preload dosing or model-based leakage correction [16,55,139]. EPI-related distortions, AIF selection, and low SNR at reduced dose are additional considerations [25,123,141,142,143].

**Dynamic Contrast-Enhanced MRI (DCE).** In DCE, a T1-weighted 2D/3D GRE sequence tracks gadolinium uptake and washout over minutes [16,144]. Signal is converted to contrast concentration using pre-contrast T1 and known sequence parameters (flip angle, TR), and pharmacokinetic models (e.g., Tofts, extended Tofts, Patlak, 2CXM) yield K^trans^, V_e_, V_p_, and K_ep_ [6,95,145]. The choice and quality of the AIF (population-averaged vs. local) and the trade-off between temporal resolution and spatial coverage/SNR are central to model stability [55,83]. B1 inhomogeneity, motion, and partial volume effects can bias parameter estimates and should be managed with calibration and motion correction [16,83,95,144].

A side-by-side summary of DSC and DCE acquisitions, models, outputs, and typical parameter ranges is provided in Table 3.

### 11.2. Acquisition and Key Parameters

**DSC**. DSC-MRI uses T2*-weighted echo planar imaging (EPI) to detect signal changes caused by susceptibility effects during the first pass of a gadolinium bolus, typically within the first minute following injection. This approach primarily evaluates signal intensity changes related to magnetic field inhomogeneities induced by the paramagnetic contrast agent within the vascular compartment [2,90,137,138]. Typical acquisition parameters include TE 30–40 ms, TR < 2 s, and voxel sizes ~2 × 2 × 5 mm^3^. Temporal resolution should ideally be <2 s to accurately capture first-pass dynamics.

**DCE**. DCE-MRI uses T1-weighted 2D or 3D gradient echo sequences [144] to monitor the temporal evolution of gadolinium uptake and washout in tissue, typically over 5–15 min [16]. Unlike DSC, DCE is sensitive to gadolinium-induced T1 shortening and provides information on tissue permeability and vascular architecture.

### 11.3. Outputs and Units

**Figure 4:**
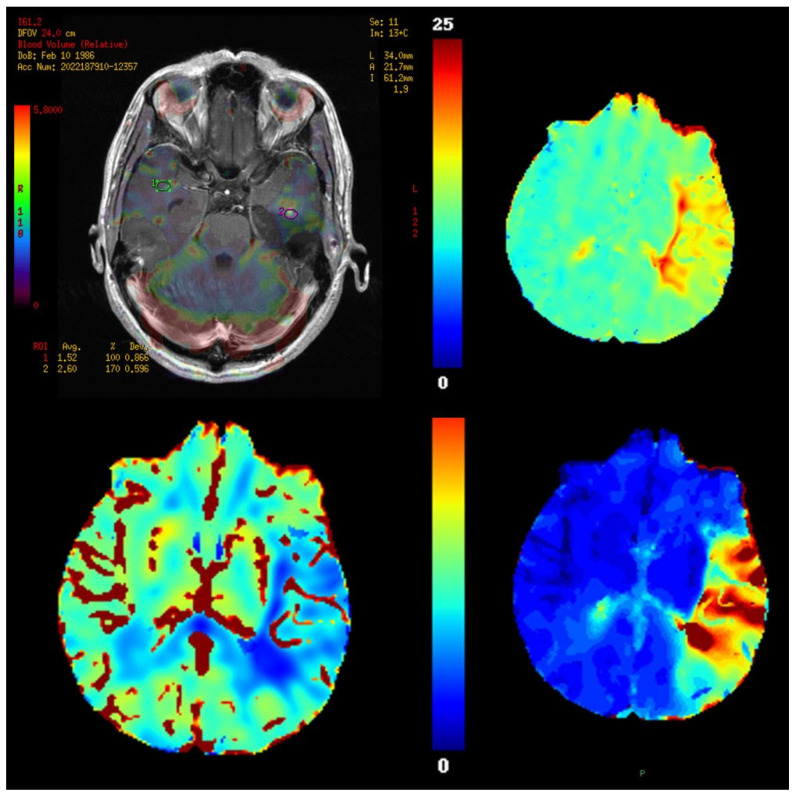
DSC-MRI perfusion parametric maps. (**Top-left**): relative cerebral blood volume (rCBV) overlaid on the structural reference, with ROIs placed in the lesion and contralateral white matter for ratio calculation. (**Top-right**): relative cerebral blood flow (rCBF) map. (**Bottom-left**): mean transit time (MTT) map. (**Bottom-right**): time-to-peak/bolus arrival delay map. Warm colors indicate higher rCBV/rCBF or prolonged transit/arrival times, whereas cool colors indicate lower values/shorter times. Maps were derived from a single-bolus T2*-weighted EPI DSC acquisition using AIF-based deconvolution (with leakage correction) and are shown with arbitrary but consistent color scales.

From DSC data, several hemodynamic parameters can be calculated, including relative cerebral blood volume (rCBV), relative cerebral blood flow (rCBF), and mean transit time (MTT). Among these, rCBV is particularly valuable, as it correlates with microvascular density, endothelial proliferation, and tumor grade [2,83,139,140]. Studies have proposed rCBV thresholds (e.g., >1.75) for distinguishing high- from low-grade gliomas. Representative DSC-MRI parametric maps—rCBV (overlay with ROIs), rCBF, MTT, and time-to-peak/bolus delay—are shown in Figure 4.

In DCE, pharmacokinetic modeling allows extraction of biologically relevant parameters such as K^trans^ (volume transfer constant between blood plasma and EES) [144,145], V_e_ (volume fraction of the EES), K_ep_ (reflux rate constant, K^trans^/V_e_), and V_p_ (plasma volume fraction). These are computed using models like Tofts, extended Tofts, Patlak, or two-compartment exchange model (2CXM), which conceptualize tissue as intravascular and EES compartments [6,16,83,95,146]. Accurate pre-contrast T1 mapping is critical for converting signal intensities to gadolinium concentration.

### 11.4. Validation and Quantification Considerations

A key limitation of DSC is its sensitivity to contrast leakage [5,139]. In high-grade tumors with a disrupted blood–brain barrier, gadolinium may extravasate into the extravascular extracellular space (EES), introducing T1-weighted effects that distort the T2*-based signal drop. To mitigate this, a pre-bolus of gadolinium is sometimes administered to saturate the EES, minimizing leakage effects during the main bolus and improving the accuracy of rCBV measurements [16,55,83]. Alternatively, mathematical leakage correction models, such as the Boxerman–Schmainda–Weiskoff method [139], can be applied.

Signal analysis in DSC often involves deconvolution techniques, with basic methods available in most commercial software. More advanced approaches, such as Bayesian deconvolution [142,143], offer greater accuracy and robustness in low SNR conditions, enabling reduced contrast doses and improved quantification [25,83,123,141]. Additionally, rCBV histogram analysis has been used to predict treatment response and clinical outcomes in high-grade gliomas.

In DCE, a major technical challenge is the estimation of the arterial input function (AIF); difficulties arise due to low temporal resolution (>3 s), partial volume effects, and voxel placement. Strategies include population-averaged AIFs, local AIFs, or dual-bolus methods with high temporal resolution [55,83].

Given the diversity of DSC/DCE inputs (AIF estimation, leakage correction, PK models, ATT), AI systems designed to handle, harmonize, and present multi-parametric outputs (e.g., rCBV/rCBF/MTT, K^trans^/V_e_/V_p_, CBF/ATT) with standardized visualization and quality flags are likely to improve robustness and clinical usability [31,32].

### 11.5. Clinical Applications

Both DSC and DCE provide valuable tools in neuro-oncology, particularly in:

- Tumor grading and characterization,
- Assessing treatment response, especially in therapies targeting angiogenesis or vascular disruption [7,9,69,85],
- Differentiating tumor progression from pseudo-progression [147,148], especially in high-grade gliomas [16,55,83],
- Evaluating brain metastases before and shortly after stereotactic radiosurgery (SRS), where perfusion and diffusion-derived parameters have shown promise in predicting early response [2,83,90].

DSC remains the workhorse of MR perfusion imaging due to its speed, robustness, and widespread clinical implementation. However, DCE offers complementary information on vascular permeability and EES dynamics, and is increasingly applied in clinical trials, particularly those involving anti-angiogenic therapies or blood–brain barrier disruption [149,150].

### 11.6. Limitations and Pitfalls

Despite their clinical utility, both techniques face limitations that hinder widespread standardization:

- Model- and software-dependent variability in DCE parameter estimation,
- AIF inaccuracies and technical demands of T1 mapping,
- Lack of universal rCBV thresholds and consistent leakage correction methods,
- Parameter fitting instability in multi-compartment models,
- Absence of digital phantoms for cross-platform validation.

### 11.7. Future Directions

Recent efforts focus on standardizing acquisition protocols [31,32], validating digital perfusion phantoms, and integrating perfusion with other quantitative modalities such as diffusion, QSM, and ASL for multiparametric imaging.

## 12. Arterial Spin Labeling (ASL)

### 12.1. Physics and Signal Model

Arterial Spin Labeling (ASL) is a noninvasive MRI perfusion technique that uses magnetically labeled arterial blood water as an endogenous tracer [151,152], eliminating the need for exogenous contrast agents. This makes ASL particularly suitable in clinical scenarios where contrast is contraindicated, such as in patients with renal insufficiency, pediatric populations, and longitudinal follow-up studies [25,83,85,96,153]. ASL enables quantitative measurement of cerebral blood flow (CBF) in physiological units (mL/min/100 g of tissue) by acquiring two sets of images: one with a labeling pulse that inverts the magnetization of arterial blood, and one control image without inversion. The subtraction of the labeled image from the control provides a perfusion-weighted map reflecting the amount of labeled blood delivered to tissue [151,154,155]. Under a single-compartment kinetic description, the label–control difference is proportional to labeling efficiency and arterial magnetization, scaled by the blood–tissue partition coefficient, and attenuated by blood T1 and arterial transit time (ATT) [154,155,156,157]. Consequently, the signal depends on several physiological and technical factors, including the T1 relaxation time of blood (~1650 ms at 3T) and ATT [154,155]; background suppression minimizes static-tissue signal and motion sensitivity [151], while calibration of labeling efficiency and a reference M0 image support absolute quantification [151,158,159]. To reduce ATT-related variability, precise timing parameters such as labeling duration, post-labeling delay (PLD), and background suppression are optimized based on the target population and clinical application [151,156,157].

### 12.2. Acquisition and Key Parameters

There are three main labeling approaches in ASL:

- Pulsed ASL (PASL): uses a short, high-powered pulse to invert a thick slab of arterial blood proximal to the imaging volume;
- Continuous ASL (CASL): applies a long, uninterrupted RF pulse and gradient field to continuously invert blood across a fixed labeling plane;
- Pseudo-continuous ASL (pCASL): the most widely used clinical approach, combines short, rapid RF pulses and gradients to simulate continuous labeling, achieving high labeling efficiency and favorable signal-to-noise ratio [83,155,158].

pCASL has been endorsed by consensus guidelines (ISMRM, QIBA) as the clinical standard [151], typically using:

- Labeling duration: ~1.5–2.0 s
- Post-labeling delay (PLD): ~1.5–2.0 s
- 3D acquisition (e.g., GRASE or spiral)
- Background suppression pulses for artifact reduction
- Optional multi-delay protocols for estimation of ATT, particularly in cerebrovascular disorders

Newer techniques are under development:

- Velocity-Selective ASL (VS-ASL): labels blood based on flow velocity, making it less sensitive to ATT variability [151];
- Time-encoded ASL (e.g., Hadamard encoding): improves temporal resolution and enables rapid acquisition of multiple PLDs [151].

### 12.3. Outputs and Quantification

ASL uses a mono-compartment kinetic model (e.g., Buxton model) to quantify CBF, assuming negligible venous return, instantaneous delivery, and constant labeling efficiency [154,155]. However, advanced modeling incorporating ATT and bolus dispersion is increasingly used in patients with altered hemodynamics [156,157].

### 12.4. Clinical Applications and Biomarkers

ASL has demonstrated increasing clinical relevance in neurology, oncology, and neuroinflammation [160,161].

Neurodegeneration. ASL studies have consistently shown reduced CBF in the posterior cingulate cortex (PCC) and precuneus in patients with Alzheimer’s disease (AD), mirroring the hypometabolic patterns observed with [18F]FDG-PET. This spatial overlap supports the tight coupling between perfusion and glucose metabolism, positioning ASL as a noninvasive surrogate biomarker of early neuronal dysfunction [81,106,162,163]. In mild cognitive impairment (MCI), hypoperfusion in temporoparietal regions has been shown to precede structural atrophy, suggesting its utility as an early marker of disease [162].

Multiple sclerosis (MS). In MS, ASL has detected perfusion abnormalities in both normal-appearing white matter (NAWM) and evolving lesions, often before changes are visible on T2-weighted MRI or signs of blood–brain barrier disruption [161,164]. ASL studies have shown:

- Reduced CBF in NAWM and cortical gray matter;
- Associations between low CBF and increased disability scores (EDSS), cognitive impairment, and atrophy;
- Distinct perfusion patterns between relapsing-remitting and progressive MS phenotypes [25,83,96].

Neuro-oncology. ASL offers a contrast-free alternative to DSC or DCE perfusion imaging. Particularly useful in pediatric patients or those with impaired renal function [161,165], ASL has been used to:

- Differentiate low-grade from high-grade gliomas, with pooled accuracy around 83%, using a CBF ratio threshold near 1.45 [16,165];
- Predict IDH mutation status in gliomas, as IDH-mutant tumors often exhibit reduced perfusion [165];
- Assess perfusion heterogeneity using histogram metrics (e.g., skewness, kurtosis), which correlate with tumor grade and progression [165].

### 12.5. Advantages, Limitations, and Future Directions

ASL’s repeatability, quantitative output, and lack of contrast requirements make it ideal for longitudinal studies, pediatric imaging, and patients with renal insufficiency [151,164]. Moreover, CBF is expressed in absolute units (mL/100 g/min), facilitating cross-center comparisons and longitudinal tracking [151,160].

However, several limitations must be addressed:

- Low signal-to-noise ratio (SNR) compared to DSC [151];
- Sensitivity to ATT variability, especially in elderly or vascular patients [157,166] (partially addressed with multi-delay ASL);
- Dependence on hematocrit, labeling efficiency, and T1 relaxation properties of blood [158,159];
- Limited spatial resolution and susceptibility to motion artifacts [167].

Ongoing efforts aim to standardize acquisition and quantification protocols, integrate ASL into multi-parametric imaging pipelines, and expand its role in precision diagnostics, particularly in neurovascular and neurodegenerative conditions [151,152,160,165,168,169].

## 13. Brain Volume Quantification

### 13.1. Scope and Overview

MRI-based brain volumetry has emerged as a fundamental tool in the evaluation of neurodegenerative and demyelinating disorders, particularly dementia and multiple sclerosis (MS). Quantitative assessment of regional and global brain atrophy provides clinically relevant biomarkers that improve diagnostic precision and help monitor disease progression [170,171].

### 13.2. Acquisition Physics and Pre-Processing

Volumetry depends on T1-weighted 3D structural MRI (e.g., MPRAGE/IR-SPGR). Accurate morphometry requires bias-field correction (B1-driven intensity non-uniformity), robust skull-strip, and partial volume handling at CSF/GM/WM interfaces. Cross-vendor harmonization (voxel size, TI/TR/TE/flip) and gradient nonlinearity correction reduce systematic bias across time and sites [27,113].

### 13.3. Acquisition and Processing Pipeline

Common approaches include:

- Voxel-based morphometry (VBM): detects regional differences in GM/WM density,
- Surface-based morphometry (SBM): estimates cortical thickness and curvature using 3D cortical meshes,
- Longitudinal analysis tools (e.g., SIENA, FreeSurfer longitudinal stream): quantify volume changes over time [172,173],
- Deep learning algorithms: allow for rapid and accurate segmentation, even in the presence of artifacts or lesions.

**Figure 5:**
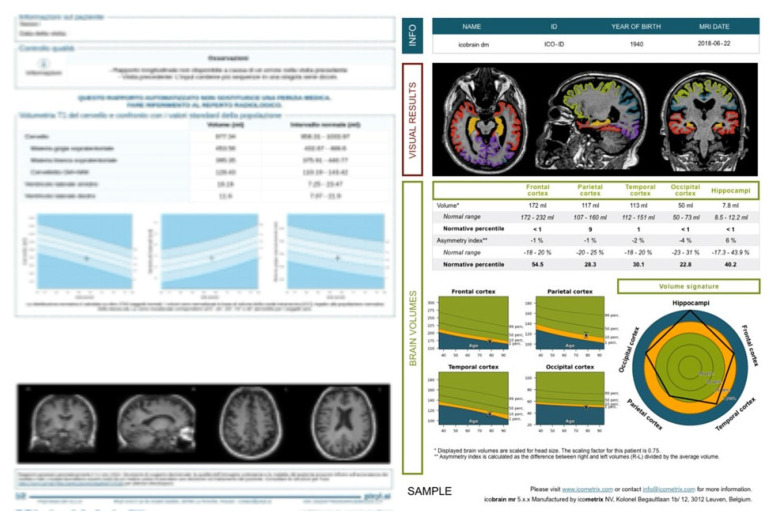
Examples of automated brain volumetry reports from commercial platforms. Each report is generated from a 3D T1-weighted MRI and illustrates: (i) segmentation overlays of cortical and subcortical structures on structural images; (ii) tables of regional/lobar and hippocampal volumes, typically normalized to intracranial volume (ICV); (iii) normative percentiles/z-scores (age- and sex-adjusted) with visual summaries (e.g., radial/ring plots); and (iv) percentile curves showing the patient’s measurement relative to a healthy reference across age and enabling longitudinal tracking. Such reports support clinical assessment (e.g., dementia work-up) by highlighting regional atrophy patterns and asymmetries. Trade names/layouts shown for illustration only; values and thresholds can vary across software and require local validation and quality control. Color legend. *Segmentation overlays (**both panels**)*: colored labels differentiate anatomical regions (software-defined palette; no direct biophysical meaning). Anatomical backgrounds are grayscale. ***Left panel** (percentile charts)*: light-blue shaded bands represent the normative 5th–95th percentile range; the thin central blue linemarks the 50th percentile; the patient’s measurement/trajectory is shown by the darker blue point/line. ***Right panel**—small “Brain volumes” graphs*: the orange band denotes <10th percentile; the green band denotes 10th–90th percentile (normative range); the thin central green line is the 50th percentile; the patient’s values are plotted in black (points/lines). Values >90th percentile lie above the green band. ***Right panel**—radial/ring “volume signature”*: the green ring depicts the normative reference profile (≈z 0), while the patient’s profile is drawn in black (polygon/trace). Sectors falling within the orange band correspond to <10th percentile.

Methodological refinements and harmonized processing pipelines continue to improve robustness across scanners and sites [174,175]. Examples of automated report outputs (segmentation overlays, regional volumes normalized to ICV, and age-adjusted normative z-scores) are shown in Figure 5.

### 13.4. Outputs and Units

From a biomarker standpoint, quantitative volumetry enhances diagnosis and risk stratification. Hippocampal volume below 3.0 cm^3^ is a known marker of AD and has been shown to predict conversion from mild cognitive impairment (MCI) to AD. Cortical thinning in the entorhinal cortex, parahippocampal gyrus, and posterior cingulate cortex is associated with early neurodegeneration, while ventricular enlargement provides complementary information in hydrocephalus or advanced disease. Normative z-scores < –1.5 are typically considered abnormal [176,177].

### 13.5. Clinical Applications—Dementia

Quantitative Volumetry in Dementia. In Alzheimer’s disease (AD) and other forms of dementia, specific patterns of regional atrophy—such as medial temporal lobe atrophy (MTA)—can aid in distinguishing between subtypes. Traditionally, these markers are assessed visually (e.g., the MTA scale), but visual evaluations are susceptible to inter-rater variability, especially early on when atrophy patterns may overlap with healthy aging [178,179].

To overcome these limitations, automated quantitative tools have been developed that compare a patient’s brain volumes with normative databases derived from healthy control populations. These methods offer multiple advantages: increased diagnostic objectivity, improved early detection of subtle atrophy, reduced dependence on subjective visual interpretation, and support from emerging AI-driven software packages [67]. Several commercial solutions now offer AI-based volumetric analysis platforms (e.g., NeuroQuant [180], FreeSurfer [172,173], VolBrain, QyScore), with the ability to compute regional volumes, cortical thickness, and normative z-scores adjusted for age and sex [181,182].

### 13.6. Clinical Applications—Multiple Sclerosis

Brain Atrophy in Multiple Sclerosis. In MS, brain atrophy reflects chronic neurodegeneration and correlates more strongly with physical and cognitive disability than lesion burden alone. Atrophy is detectable from the earliest stages, including clinically isolated syndrome (CIS), and tends to progress steadily throughout the disease course [22,55,183].

Key volumetric markers in MS include: annual whole-brain atrophy rates of 0.5–1% (vs. 0.1–0.3% in healthy individuals), a commonly accepted threshold for pathological brain volume loss of ≥0.4%/year, and pseudoatrophy (treatment-induced resolution of inflammatory edema) that should not be mistaken for true neurodegeneration [22,184].

Regional analysis further refines our understanding of atrophy-related dysfunction: deep gray matter (GM) structures (thalamus, putamen, caudate) are frequently affected early; cortical GM atrophy tends to develop later and is more prominent in progressive MS; GM atrophy is a stronger predictor of long-term disability than white matter (WM) atrophy; and GM loss is linked to conversion from CIS to clinically definite MS (CDMS) [55,185].

Clinical correlations include: thalamic and central GM atrophy associated with cognitive dysfunction, fatigue, and gait impairment; cortical thinning in motor/parietal regions related to fine hand coordination; and periventricular lesion load correlating with cortical GM loss, possibly via CSF-mediated neurodegenerative processes [22,186].

Longitudinal MRI studies show that baseline brain volume, percent brain volume change (PBVC), and black-hole lesion volume are among the most robust predictors of future disability progression. Quantification tools such as SIENA (FSL), longitudinal FreeSurfer [172,173] pipelines, and deep learning segmentation frameworks (e.g., nnU-Net) are increasingly used in research and, more recently, clinical settings.

### 13.7. Validation and Repeatability

Inter-software agreement remains suboptimal. Studies have reported differences of up to 10–15% in hippocampal and cortical volume estimates across platforms, largely due to variations in segmentation algorithms, reference atlases, and intracranial volume correction [68]. Thus, while intra-rater reproducibility is generally high, consistency between software outputs remains moderate, particularly in diagnosis-specific interpretation. Discrepancies in intracranial volume measurement and regional labeling may influence diagnostic conclusions, especially when borderline values are involved; clinics are therefore advised to independently evaluate and validate volumetric software before integration into clinical workflows [185].

### 13.8. Limitations and Pitfalls

Volumetric quantification faces several challenges: variability in acquisition protocols (scanner type, field strength, MPRAGE vs. SPGR), motion artifacts, signal dropouts, partial volume effects, undersegmentation in severe atrophy or enlarged ventricles, and lack of universally accepted normative datasets across ethnicities and age groups. These issues contribute to moderate cross-software agreement and must be considered when interpreting borderline volumetric findings [89,90].

### 13.9. Future Directions

As AI-driven volumetric tools become more accessible, their potential in personalized neurology continues to grow. When rigorously validated, brain volumetry provides a powerful, objective complement to conventional radiological assessment. In dementia, it improves diagnostic accuracy and reduces inter-observer variability. In MS, it offers sensitive measures of disease burden and long-term prognosis, often outperforming conventional lesion metrics [22,55,67,68].

## 14. Conclusions

Quantitative MRI (qMRI) represents a transformative advancement in neuroimaging, offering unprecedented sensitivity and specificity in the evaluation of tissue microstructure, physiology, and pathology. While qMRI is increasingly used to interrogate cortical plasticity, learning-related remodeling, and pediatric development, these areas are deliberately out of scope here to maintain a clinically focused synthesis on adult disease. Future work can extend this structured framework to those domains [105].

Techniques such as T1 and T2 relaxometry, diffusion imaging, quantitative susceptibility mapping (QSM), magnetization transfer (MT) imaging, perfusion imaging, and volumetry have proven to be powerful tools for deepening our understanding of central nervous system (CNS) disorders, including neurodegenerative diseases, inflammatory conditions, and brain tumors.

Despite this potential, qMRI has not yet reached full clinical maturity [100]. Several limitations currently restrict its routine application, including:

- The lack of standardized acquisition protocols across vendors and platforms,
- Limited availability of robust, validated software for map reconstruction and biomarker extraction,
- Absence of large normative datasets and clinically validated pathological cut-off values,
- The need for multicenter clinical validation studies directly comparing qMRI metrics to established clinical, histological, or molecular outcomes.

Furthermore, while brain-focused qMRI applications have matured considerably, their extension to spinal cord imaging remains technically challenging. Limitations such as spinal motion, physiological pulsatility, and reduced cross-sectional area require:

- Motion-compensated acquisition strategies and physiological gating,
- Dedicated hardware (e.g., optimized phased-array coils),
- Advanced software for region-of-interest (ROI) localization and signal modeling.

In parallel, the growing use of quantitative imaging biomarkers (QIBs) supports the broader movement toward personalized and precision medicine. QIBs—defined by their objectivity, repeatability, and biological relevance—are increasingly used not only for diagnosis but also to guide prognosis and monitor treatment efficacy. When fully validated, they may serve as surrogate endpoints in clinical trials and inform risk stratification and treatment algorithms [19].

To achieve widespread clinical integration, qMRI must also embrace quantitative metrology, ensuring that metrics demonstrate technical performance in terms of bias, precision, and reproducibility. Initiatives such as RSNA QIBA and EIBALL have proposed methodological frameworks for technical validation and standardization, which are essential for regulatory approval and cross-site consistency.

Recent advances are accelerating spinal qMRI: reduced-FOV EPI and motion/CSF-pulsation management enable more reliable DTI in cervical cord (e.g., degenerative cervical myelopathy), with FA/MD/RD correlating with impairment and subclinical degeneration; MT/MTsat and MWF extend myelin-sensitive mapping; preliminary QSM/SWI work targets hemorrhage and disk/degenerative changes. Key challenges remain (small cross-section, B0/B1 inhomogeneity, susceptibility interfaces, motion), but multicenter harmonization and dedicated coils/sequences are improving robustness, supporting near-term translation in MS, SCI, and DCM [105,106,107,108].

Another key enabler is the development of AI-driven pipelines, which promise to streamline post-processing, improve the robustness of parametric mapping, and facilitate the detection of subtle disease patterns. AI also enables integrative modeling by combining qMRI data with genomics, cognitive scores, and fluid biomarkers, ultimately enhancing clinical decision support. Emerging AI/ML tools already address key bottlenecks: (i) denoising (self-supervised and patch-based) for diffusion/ASL to improve SNR without repeat scans; (ii) motion and distortion correction (navigator-less retrospective motion estimates; learned susceptibility-distortion correction for EPI); (iii) harmonization across vendors/sites with outlier-aware domain adaptation; (iv) QSM inversion with learned regularizers that preserve edges and provide uncertainty maps; (v) perfusion QC (automatic AIF detection, leakage-aware model selection) and multiparametric fusion (rCBV/K^trans^/CBF with standardized color scales); (vi) automated report generation (normative z-scores, longitudinal deltas) exported as DICOM-SR for PACS integration. These examples illustrate how AI/ML can reduce noise/artifacts and deliver consistent, interpretable outputs at scale [130,131,132].

To translate qMRI into routine care, AI should target four concrete needs: (i) metric coherence, enforcing internal consistency across modalities (e.g., R1 = 1/T1), unit checks, cross-modality co-registration with resolution matching, and outlier-aware harmonization across vendors/sites; (ii) robust visualization, delivering standardized, perceptually uniform maps with linked views, anatomical overlays, and uncertainty/QC layers; (iii) automatic QC and failure detection (motion, bias fields, EPI distortions, leakage effects); and (iv) report generation with normative z-scores, longitudinal deltas, and multiparametric summaries integrated into PACS/DICOM-SR [31,32,67].

Finally, qMRI is poised to evolve from a set of research techniques to a clinically robust imaging framework. Through interdisciplinary collaboration among clinicians, physicists, data scientists, and regulatory agencies, and with the support of ongoing multicenter efforts, qMRI can become a cornerstone of modern neurodiagnostics—enabling early detection, disease characterization, and precision-guided therapy across a broad spectrum of neurological conditions.

## Figures and Tables

**Table 1:** Disease-centric mapping of qMRI biomarkers (adult brain) (p.u., “percentage units,” denotes software-specific unitless MTsat scale) [23,37,38].

Disease/Condition	Most Relevant qMRI Biomarkers (Examples)	Typical Readouts (Units)	Representative Uses
Multiple sclerosis (MS)	Myelin/MT (MWF, MTsat), T1/R1, DTI (FA/RD), QSM (rim-positive), volumetry (GM/thal)	MWF (%), MTsat (p.u.), T1 (ms), R1 (s^−1^), FA/RD (–/mm^2^/s), χ (ppm), volumes (cm^3^)	Demyelination vs. repair; lesion staging; disability risk; progressive disease monitoring
Dementia/AD	Volumetry (hippocampus/cortex), ASL-CBF, QSM (deep nuclei iron)	cm^3^; cortical thickness (mm); CBF (mL/100 g/min); χ (ppm)	Early diagnosis; subtype patterns; progression tracking
Neuro-oncology (gliomas/metastases)	DSC rCBV, DCE K^trans^/V_e_, ADC, QSM (calcification vs. hemorrhage)	rCBV (ratio), K^trans^ (min^−1^), V_e_ (–), ADC (mm^2^/s), χ (ppm)	Grading; pseudo-progression vs. progression; early response (SRS/anti-angiogenic)
Ischemic stroke	DWI/ADC, DKI MK, ASL-CBF, DSC delay/MTT	ADC (mm^2^/s); MK (–); CBF (mL/100 g/min); MTT (s)	Core/penumbra; tissue-at-risk delineation
TBI	SWI/QSM (microbleeds), DTI (FA/RD), volumetry	χ (ppm); FA/RD; cm^3^	Diffuse axonal injury; microhemorrhage burden; prognosis
Spinal cord/DCM	DTI (FA/MD), MT/MTsat, MWF	FA/MD; MTsat; MWF (%)	Subclinical degeneration; severity; outcome prediction

**Table 2:** qMRI modalities at a glance (brain). Typical acquisitions/readouts, approximate clinical scan times, primary outputs (units), strengths, and common limitations for the modalities reviewed. Values indicate typical clinical ranges and may vary with vendor/protocols.

Modality	Typical Acquisition	Approx. Time	Primary Outputs (Units)	Strengths	Common Limitations
T1 relaxometry	IR/MP2RAGE; VFA (B1-corrected); SyMRI	~4–8 min	T1 (ms), R1 (s^−1^)	Myelin/sclerosis sensitivity; whole-brain maps	B1/MT bias; sequence heterogeneity
T2 relaxometry/MWF	MESE/GRASE; mcDESPOT	~4–8 min	T2 (ms), MWF (%)	Myelin-related specificity	Stimulated echoes; ill-posed multi-component fits
MT (MTR/MTsat/qMT)	GRE with MT prep; multi-parametric MT	~4–7 min	MTR (p.u.), MTsat (p.u.), qMT params	Myelin/macromolecule sensitivity	B1 dependence; vendor diversity
Diffusion (DWI/DTI/DKI/NODDI)	EPI with ≥30 dirs; multi-b shells	~3–10 min	ADC/FA/MD; MK/NDI/ODI	Microstructure; tractography	EPI distortions; motion/eddy; model dependence
SWI/QSM	3D multi-echo GRE	~4–7 min	SWI (qual.), χ (ppm)	Veins/iron; calcification vs. hemorrhage	Ill-posed inversion; regularization trade-offs
Perfusion (DSC/DCE/ASL)	T2* EPI (DSC); 3D GRE (DCE); pCASL	~2–3/5–15/4–6 min	rCBV/rCBF/MTT; K^trans^/V_e_/V_p_/K_ep_; CBF/ATT	Vascular density/permeability/flow	Leakage/AIF/ATT; SNR; model variance
**Volumetry**	3D T1 (MPRAGE/SPGR)	~4–6 min	Regional volumes (cm^3^), thickness (mm)	Objective atrophy metrics	

**Table 3:** Contrast-based perfusion MRI sequences (DSC vs. DCE). Side-by-side summary of Dynamic Susceptibility Contrast (DSC) and Dynamic Contrast-Enhanced (DCE) MRI. DSC uses T2*-weighted EPI (temporal resolution < 2 s; duration ~2–3 min) to derive rCBV, rCBF, and MTT, reflecting microvascular density; principal limitations are contrast leakage and susceptibility/EPI artifacts. DCE uses T1-weighted 3D GRE (temporal resolution ~4–6 s; duration ~5–15 min) to estimate K^trans^, V_e_, K_ep_, V_p_, indexing capillary permeability; limitations include AIF estimation and modeling variability.

Feature	DSC	DCE
Sequence	T2*-weighted EPI	T1-weighted 3D GRE
Key Parameters	rCBV, rCBF, MTT	K^trans^, V_e_, K_ep_, V_p_
Temporal Resolution	<2 s	~4–6 s
Duration	~2–3 min	~5–15 min
Sensitivity	Microvascular density	Capillary permeability
Limitations	Leakage effects, susceptibility	AIF estimation, modeling variability

## Data Availability

No new data were created or analyzed in this study. Data sharing is not applicable to this article.
